# Antibodies and tuberculosis

**DOI:** 10.1016/j.tube.2016.08.001

**Published:** 2016-12

**Authors:** Ashley J. Jacobs, Juthathip Mongkolsapaya, Gavin R. Screaton, Helen McShane, Robert J. Wilkinson

**Affiliations:** a Department of Medicine, Imperial College London, W2 1PG, United Kingdom; b Clinical Infectious Diseases Research Initiative and Department of Medicine, Institute of Infectious Diseases and Molecular Medicine, University of Cape Town, Observatory 7925, South Africa; c The Jenner Institute, University of Oxford, OX3 7DQ, United Kingdom; d The Francis Crick Institute, London NW1 2AT, United Kingdom

**Keywords:** Tuberculosis, Antibodies, Humoral immunity, Vaccine

## Abstract

Tuberculosis (TB) remains a major public health problem internationally, causing 9.6 million new cases and 1.5 million deaths worldwide in 2014. The Bacillus Calmette-Guérin vaccine is the only licensed vaccine against TB, but its protective effect does not extend to controlling the development of infectious pulmonary disease in adults. The development of a more effective vaccine against TB is therefore a pressing need for global health. Although it is established that cell-mediated immunity is necessary for the control of latent infection, the presupposition that such immunity is sufficient for vaccine-induced protection has recently been challenged. A greater understanding of protective immunity against TB is required to guide future vaccine strategies against TB.

In contrast to cell-mediated immunity, the human antibody response against *M.tb* is conventionally thought to exert little immune control over the course of infection. Humoral responses are prominent during active TB disease, and have even been postulated to contribute to immunopathology. However, there is evidence to suggest that specific antibodies may limit the dissemination of *M.tb*, and potentially also play a role in prevention of infection via mucosal immunity. Further, antibodies are now understood to confer protection against a range of intracellular pathogens by modulating immunity via Fc-receptor mediated phagocytosis. In this review, we will explore the evidence that antibody-mediated immunity could be reconsidered in the search for new vaccine strategies against TB.

## 1 Introduction

Tuberculosis (TB) is the leading cause of death from bacterial infection worldwide, with 9.6 million cases and 1.5 million deaths in 2014 [1]. The Bacillus Calmette–Guérin (BCG) vaccine was introduced to prevent disease during the mid-20th century but, despite widespread coverage, has failed to control the spread of TB in high burden areas [1]. The continuing rise of infections in such areas despite vaccination is in part due to the BCG vaccine's variable efficacy in preventing the development of adult pulmonary TB [2], [3]. Expectoration of *Mycobacterium tuberculosis* (*M.tb*), the causative agent of TB, by adults with active pulmonary disease drives the ongoing transmission of the disease. There is an urgent need for a more effective vaccine against TB, as the *WHO Stop TB* collaboration's goal of eliminating TB as a threat to global health cannot be reached even with optimal implementation of current interventions [4].

The existence of natural immunity against TB is supported by the observation that nine out of ten individuals appear able to control infection with *M.tb* in a state of clinical latency [5]. However, the precise immune requirements needed for this immunity are incompletely defined, and hence the immune response to target by vaccination remains elusive [6]. The contribution of cell-mediated immunity (CMI) here has been firmly established in past decades, and it is thus reasonable that a vaccine against TB should induce a CD4^+^ T-cell response against immunodominant T-cell antigens [6]. The MVA85A is one such vaccine and was recently tested in two landmark efficacy trials [7], [8]. Despite demonstrating protection in some animal models, and inducing antigen specific CD4+ T-cells, MVA85A was unable to add to the protection assumed to be provided by BCG [7], [8]. Many candidate vaccines against TB target a similarly narrow immune repertoire, and thus the disappointing outcome of the MVA85A trials has provided impetus to explore a wider range of immune responses in protection against TB [9], [10].

Antibody-mediated immunity (AMI) is one such approach. As *M.tb* is a facultative intracellular pathogen, it has been postulated that antibodies either have no protective benefit or may even contribute to immunopathology in active disease [11]. Surmounting this presumed lack of functional antibodies in TB presents a substantial challenge for the next generation of vaccines against TB, as antibody titre and specificity remains the predominant correlate of vaccine-induced immunity for many other diseases [12]. Even in diseases where antibodies produced during infection fail to confer protection, vaccines have been designed to induce antibodies capable of protecting from disease. Such ‘synthetic’ or non-natural immunity utilizing antibodies may present a novel testable vaccine hypothesis against TB. Here we will explore the recent expansion of evidence that a role for antibodies in immunity is worthy of consideration in designing future vaccine strategies against TB.

### 1.1 Humoral immunity during natural infection with TB

#### 1.1.1 Variation in human antibody responses against *M.tb*

It has long been known that natural infection induces the formation of antibodies against *M.tb*. In the late 19th century it was thought that antibodies formed in inoculated animals would be able to treat infection in patients as this approach was successful in pneumococcal disease [13]. The inconsistent trial results that followed were an early clue to the complexity of the antibody response against *M.tb* [13].

Studies following on from these original trials demonstrated that 90% of TB patients have raised titres of serum immunoglobulin against mycobacterial antigens at the time of clinical presentation [14]. However, the antigens targeted by individual patients vary widely, as one study showed that out of a panel of ten culture filtrate proteins secreted by *M.tb*, no single antigen was universally recognized by serum from patients with active TB [14]. The correlation between antibody responses and active TB disease led to investigation of antibodies as diagnostic markers rather than a therapeutic strategy, but these efforts were discouraged by the WHO in 2012, due to suboptimal sensitivity and specificity in studies [15]. It should be noted however that this recommendation was only directed towards the diagnostic use of current commercially available tests and not towards investigation into the function of antibodies in immunity against TB as a whole. Many factors influence the development of antibodies during the course of infection including latency, stage of infection, HIV and host genotype as summarized in Table 1.

#### 1.1.2 Markers of humoral immunity in recent studies

A consistent finding in whole blood transcription studies in active TB, spanning geographical locations and in HIV-1 co-infection, is the up-regulation of the high-affinity antibody receptor FCγR1A [16], [17], [18]. Fcγr1A binds antibody principally of the IgG1 and IgG3 subtype and is expressed mainly in macrophages and dendritic cells [19]. The expression of complement C1q, which forms immune complexes with immunoglobulin, is also elevated during active TB and is associated with increased disease severity [17], [20]. Further analysis of transcription studies have shown TRIM21, a recently identified intracellular antibody receptor, to also be elevated in disease [21]. This receptor activates a cascade of signalling pathways known to be important in the control of adenovirus and salmonella infection by activation of NFκB and proteasomal degradation pathways [22] What function TRIM21 may play in TB is however speculative at present [22].

Humoral immunity is also stimulated at the site of disease: pulmonary TB is associated with an increase in the Th2 cytokines IL-4, CCL-4 and SOCS3 in bronchoalveolar lavage (BAL) fluid taken from patients with active pulmonary TB [23]. IL-4 levels in sputum correspond to increased bacterial load and antibody levels in serum [24]. Antibodies are present in the sputum of patients with active pulmonary TB as shown by mass spectroscopy [25]. This polarization towards a humoral immune response has previously been hypothesized to represent immune evasion by the skewing of host responses away from a protective Th1-mediated response [23]. Despite the variable AMI response in individual patients and the association of antibody responses with the clinical state of active disease, there are multiple clinical scenarios in which AMI may be protective against *M.tb*.

### 1.2 Antibody-mediated protection in humans

#### 1.2.1 Humoral immunodeficiency and risk of TB

Clinical observations have offered considerable insights into understanding natural immunity to *M.tb* infection. Clear examples of these findings include the observation that HIV-infected patients are at greater risk of developing TB, and the loss of CD4^+^ cells correlates to an increasing risk of disseminated disease [26]. Genetic susceptibility to mycobacterial disease is well described and is typically seen in loss of functionality in the IL-12, STAT1 and IFN-γ pathways [27]. If humoral immunity plays a role in mediating protection against TB, we could reasonably expect to observe patients with attenuation of antibody responses to be at greater risk of developing active infection.

##### 1.2.1.1 Clinical observations suggesting little role for antibodies in TB immunity

Meta-analysis in diseases leading to a loss of antibody such as X-linked agammaglobulinemia or common variable immunodeficiency (CVID) have not shown an increased risk of active TB, although these findings may be confounded by variable exposure to *M.tb* and intravenous immunoglobulin (IVIG) replacement [28]. In patients taking Rituximab, an anti-CD20 monoclonal antibody that depletes populations of naïve B-cells, there does not appear to be an increased risk of TB reactivation [29]. This is in contrast to patients taking anti-TNF-α monoclonal antibodies for rheumatic conditions who are at increased risk of reactivation TB [30].

##### 1.2.1.2 Clinical observations in favor of a role for antibodies in TB immunity

Case reports of endobronchial tuberculosis in X-linked agammaglobulinemia, cutaneous mycobacterial infection in selective IgA deficiency and extra-pulmonary TB in autosomal recessive hyper-IgM syndrome have been reported [31], [32], [33]. Autosomal recessive hyper-IgM syndrome prevents the hyper-mutation of specific immunoglobulins, with intact cellular immunity, unlike X-linked forms where T-cell activation is impaired [31], [32], [33]. Although these appear in isolation, they do suggest a contributing role of antibodies in immunity against mycobacterial infection.

#### 1.2.2 Antibodies modulate severity of TB disease

An alternative role for antibodies could be to modulate the course of *M.tb* infection. Disseminated TB has a higher mortality rate than pulmonary TB, and the finding that children without detectable antibodies against LAM, or to extracts of mycobacteria rich in LAM, are at greater risk of disseminated TB is of note [34]. Corroborating this finding is that the period of greatest risk for disseminated disease corresponds to a trough in antibody titres against LAM between the ages of six months and three years [34]. A similar phenomenon was observed for antibodies against the 38 kDa antigen of *M.tb* [35]. A lack of antibodies against this protein is associated with the development of extra-pulmonary TB in children or TB meningitis in adults, and ELISA detecting this antigen has been used to diagnose TB from CSF [35]. Furthermore, Ziegenbalg et al. screened patients for antibodies against mycobacterial membrane vesicles containing an abundance of surface proteins and glycolipids, and the three patients in the cohort with disseminated disease lacked antibodies against these antigens [36].

Recently, higher titres of IgG against Ag85A have been shown to associate with reduced risk of developing active disease in an infant case-control study [37]. This raises the possibility that anti-mycobacterial antibodies assist in the containment of initial infection with *M.tb* in humans. The presence of IgG against Ag85A has elsewhere been associated with decreased cavitation and a greater chance of sputum clearance of *M.tb* in a cohort of Mexican patients [38] The development of clinically defined AIDS is associated with waning antibody titres against *M.tb* antigens in purified protein derivative (PPD) – suggesting that the risk for severe disease in this population may not be exclusively due to loss of CD4^+^ cells [39]. IgA against α–crystallin (Acr) and HrpA are associated with improved markers of disease severity at the time of admission, with lower albumin and CRP levels seen in patients with higher titres of antibodies against these antigens [40]. These findings suggest that a vaccine eliciting such antibodies may prevent extrapulmonary TB and thus reduce mortality and morbidity from TB.

#### 1.2.3 Antibodies in prevention of infection with *M.tb*

It has been conjectured that if protective antibodies in TB are present in humans, that they may have a role in preventing the initial acquisition of infection with *M.tb* [41]. The discovery of individuals in high-exposure settings that do not have a detectable T-cell response to *M.tb* antigens by tuberculin skin testing (TST) or *ex vivo* stimulation has triggered interest in the search for individuals with unusually effective immune responses against TB [42]. This phenotype was established with the finding that 20% of individuals from a hyper-endemic area remain unreactive to TST, without signs of infection, representing possible natural resistance to infection [42]. Genome-wide linkage survey performed on these patients revealed two loci potentially responsible for this phenotype on chromosomes 11p14 and 5p15 linked to a TNF-α response element [42]. A similar cohort of nurses in a TB ward that remained TST-negative despite years of exposure underwent antibody repertoire sequencing with next-generation sequencing [43]. Here an IgA gene rearrangement of VH3-23-D3-3-J4 was predominant amongst those nurses who remained TST-negative and did not develop active disease compared to their TST reactive colleagues [43]. An earlier study of nurses exposed to TB revealed a strong antibody response against a non-immunodominant epitope of Acr [44]. A possible conclusion of such studies is that these individuals produce antibodies against *M.tb* that prevent or help resolve infection despite high exposure to the bacillus

Further suggestion for the role of antibodies in individuals with a long duration of close contact to active TB patients that remain TST-negative is that these individuals display increased levels of IgG against *M.tb,* and that their serum is able to block or enhance stimulation of autologous T-cells in PBMC by purified protein derivative (PPD) [45]. Individuals highly exposed to TB produce high levels of IgG against *M.tb* PPD, and antibody levels against mycobacterial surface polysaccharides in healthy individuals from India were found to match those in patients with active disease [46], [47]. Although these findings are suggestive of a role for antibodies in protection, prospective studies would demonstrate if heightened antibody responses associate with long-term reduction in risk for developing active or latent TB [48]. If this is found to be the case, it would strongly support existing studies that the induction of AMI by vaccination offers a mechanism whereby the initial acquisition of infection can be prevented.

### 1.3 The role of opsonizing antibodies and antibody isotypes against *M.tb*

*M.tb* is one of the most successful human pathogens, owing to millennia of coevolution, and as such, has an array of mechanisms to circumvent the establishment of a protective immune response [49]. The intracellular niche of *M.tb* allows it to avoid antibodies for parts of it lifecycle, but additional defences mechanisms against AMI may be present and provide an explanation for why antibodies do not appear to protect against active TB. If understood, these mechanisms could potentially by circumvented by vaccination to produce protective antibodies.

#### 1.3.1 Sites of interaction between antibodies and *M.tb*

A major criticism against the role of antibodies in immunity against *M.tb* is the question of where antibodies could interact with sufficient effect against the bacillus. Antibodies are present in both the upper and lower lung fields – IgA predominates in the upper airway and IgG in the lower airway [12]. Human infection with *M.tb* does induce IgG and IgA formation against *M.tb* antigens in BAL fluid, although the extent of interaction between antibodies in the respiratory mucosa and inhaled *M.tb* is essentially unknown [50], [51].

There is clear indication that antibodies are able to bind *M.tb* antigens at the site of infection. B-cells surround the granuloma in non-human primates and these cells display an activated antibody-secreting phenotype [52]. The lung tissue and draining lymph nodes of the rhesus macaques in this study were also enriched for plasma cells and immunoglobulin against the *M.tb* antigens CFP-10 and ESAT-6 [52]. In humans, activated *M.tb*-specific B-cells are found in the pleural fluid in patients with pleural TB [53]. Such antibody-secreting B-cells in the granuloma may secrete immunoglobulin that interacts with extracellular bacilli. *M.tb* is extracellular during reinfection of host cells as well as at the time of expectoration, and the guinea pig model has also shown that the necrotic core and acellular rim of the granuloma also contains significant numbers of extracellular bacilli [54]. Given the robust signal of FcyR pathway activation in active TB transcriptomic signatures, these antibodies could serve to bind free *M.tb* antigens or the bacillus itself and beneficially modulate macrophage activation.

#### 1.3.2 Surface binding antibodies against *M.tb*

Vaccines against bacterial pathogens typically induce antibodies that are able to opsonize bacteria and initiate complement-mediated lysis and uptake into neutrophils for destruction [12]. Even in intracellular bacterial infections such as *S. typhi*, surface-binding antibodies are able to trigger killing by opsonophagocytosis during the extracellular phase of their lifecycles [55]. *M.tb* presents the substantial challenge of a lipid-rich cell membrane and less potential exposure to antibodies via haematogenous spread. However, in an experimental of model *Cryptococcus neoformans*, a fungal pathogen with a similar waxy capsule, antibodies are able to bind surface-expressed antigens and provide protection by altering fungal gene expression and increasing sensitivity to antifungal drugs [56], [57].

It is therefore notable that active tuberculosis seems to stimulate an antibody response predominantly against secreted antigens rather than against surface-exposed antigens. Kunnath-Velayudhana et al. compared serum binding to a TB protein microarray which represented linearized copies of 1200 proteins, allowing determination of the antigenic repertoire of *M.tb* in patients with active TB disease. The proteins recognized by these individuals was restricted to 0.5% of the *M.tb* proteome, and greatly enriched for secreted and cytosolic antigens in comparison to healthy controls [58]. Antibodies binding these groups of antigens would likely not be able to directly bind *M.tb* during any extracellular periods.

More recent studies from other groups have since improved on the expression systems to represent a broader range of proteins, still demonstrating a narrow range of antigens targeted during infection compared to the total proteome of *M.tb* [59], [60]. Further, the development of active TB in humans is associated with a drop in avidity of antibodies that are able to directly bind the surface of live bacilli [61]. Antibodies against secreted proteins and lysate preparations increase in avidity and titre in comparison to controls, again demonstrating that *M.tb* strongly induces humoral immunity but potentially not antibodies that are able to opsonize it [61]. Vaccine induction of antibodies capable of opsonizing *M.tb* may therefore be a potential approach to harnessing AMI in TB.

#### 1.3.3 Antigenic variation in known targets of humoral immunity

The existence of functional antibodies recognizing *M.tb* surface antigens would be further supported by evidence for immune evasion by antigenic variation in these targets. Immunodominant T-cell epitopes of *M.tb* are postulated to be hyperconserved, suggesting that recognition of these epitopes by host T-cells may favor bacterial survival and transmission [62]. MPT64 is a known B-cell surface and secreted antigen and polymorphisms in clinical isolates predominately affected B-cell epitopes, with 85.71% of mutations occurring in B-cell epitopes [63], [64]. Mutations in this protein may affect both its structure and surface expression [63], [64]. The PE_PGRS33 protein is also immunogenic and forms part of the PE/PPE complex involved in pathogenesis, with 84 of 123 clinical isolates showing sequence variation compared to *M.tb* H37Rv [65], [66]. These findings lend further credence to the hypothesis that surface antigens are under evolutionary pressure from the host immune system and may be epitopes of functional antibodies in the human host.

#### 1.3.4 The role of antibody subclasses in protection

Antibody subclass may also be a factor in the apparent lack of protection from antibodies during natural TB infection, as subclasses have variable roles in facilitating interaction with other immune cells [67]. IgG1 and IgG3 are the predominant human antibody subclasses formed against TB [68]. IgG1, but not IgG3, from TB patients is able to stimulate release of TNF-α production from primary monocytes [69].

In murine models of *C. neoformans* infection, IgG3 is the predominant isotype produced during infection but antibodies of this isotype decreased survival whereas isotype switching to IgG1 conferred protection [70]. The host-pathogen interactions of other intracellular organisms demonstrate similar findings [71]. Antibodies against the surface of *Histoplasma capsulatum* are protective in mouse models but these proteins are not strongly immunogenic as evidenced by the finding that passive transfer of immune serum has no effect in models of lethal histoplasmosis [72]. It is plausible then that large amounts of non-functional or even pro-inflammatory antibodies are produced in the progression of active TB disease, but that this response can be altered by vaccination.

### 1.4 Potential mechanisms of AMI in TB

#### 1.4.1 Experimental models

Passive transfer of serum studies in mouse models have been used to broadly assess the effects of anti-mycobacterial antibodies, and results both supportive and non-supportive of a protective role have been recorded [13]. Experiments seeking to demonstrate the necessity of B-cells in TB have shown conflicting results and have been comprehensively reviewed elsewhere [73]. B-cells may well play a role in granuloma formation and amelioration of inflammatory pathology, but these effects must be isolated from those occurring as a consequence of the action of antibody.

##### 1.4.1.1 Evidence against the role of antibodies in immunity

In mice with normal B-cell counts but unable to secrete immunoglobulin due to a germline AID mutation, an increased burden of bacilli was observed in the lung and spleen upon mycobacterial challenge [74]. This bacterial burden was not abrogated by the infusion of serum from control mice naive to mycobacterial exposure. Thus the protective effect was concluded to be mediated by cytokines such IL-10 secreted by B-cells, rather than antibodies [74]. In the same study, uMT mice lacking both B-cells and immunoglobulin were not more susceptible to infection than wild-type mice. However, a limitation of this study is its inability to disprove a role for specific antibodies against *M.tb*, as serum from non-immunized healthy mice was used to replete antibodies in the AID mutation mice [74].

##### 1.4.1.2 Evidence in favor of a role for antibodies in immunity

In contrast to these findings, Kozakiewicz et al. [75] found that B-cell deficient mice responded with neutrophilia after high dose inoculation with H37Rv, and that this neutrophilia could be reversed by administration of sera from infected C57BL/6 wild-type mice. High infectious doses are a cause of neutrophilia, a marker of immunopathology, and thus the abrogation of this response by immune sera demonstrates that antibodies specific to *M.tb* do contribute to protection.

Severe combined immunodeficiency mouse model (SCID) mice are highly susceptible to infection with *M.tb* [76], In *M.*tb infected mice with partially treated infection, infusion of serum from immunized mice reduces bacillary load 100-fold and pulmonary infiltration by 3-fold [76]. Given the lack of T-cells in these mice, the mechanism of protection here is likely the role of antibody in immune serum [76]. These studies suggest a role for only anti-mycobacterial antibody containing serum in protection against TB independent of a B-cell mediated effect. Immune serum appears to mediate different effects compared to serum from non-immunized mice, most likely due to higher titres of antibodies against mycobacterial antigens.

#### 1.4.2 Antibodies modulate *M.tb* and macrophage interaction and enhance CMI via FcR-mediated phagocytosis (Figure 1)

**Figure 1:**
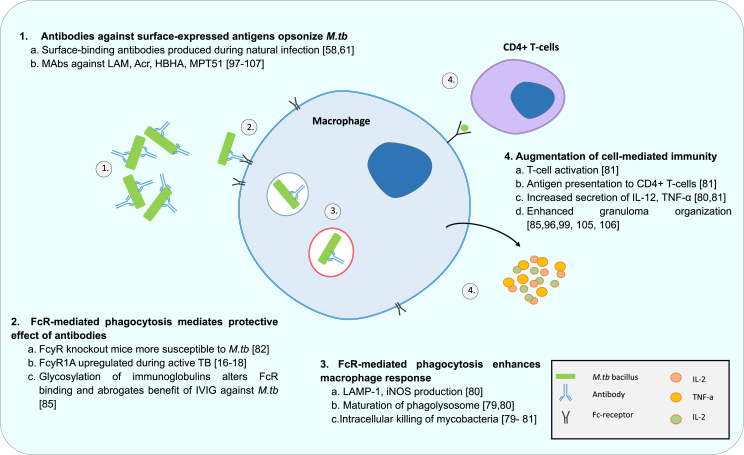
Antibodies modulate *M.tb*-macrophage interaction via FcR-mediated phagocytosis.

Antibodies are now also understood to augment CMI and reduce survival of intracellular pathogens via effector functions of the Fcγ receptor (FcR) (Figure 1) [77]. As previously established, CMI is necessary for the containment of *M.tb* in latent infection [6]. FcR-mediated phagocytosis of antigen concentrates antigen, improves phagolysosomal fusion and enhances peptide presentation to T-cells via MHC-II molecules [19]. FcR engagement is critical to the control of other intracellular pathogens such as chlamydia, and therefore presents a potential pathway whereby antibodies influence immunity against *M.tb* [78].

In one of the original studies investigating the effects of opsonisation on uptake of *M.tb*, Hart et al. [79] demonstrated in 1975 that immunized rabbit serum enhanced phagolysosomal fusion in *M.tb* infection by comparing the fate of opsonized and non-opsonized phagocytosed bacteria in phagocytes using electron microscopy (EM). Although greater phagolysosomal fusion did not have an effect on viable intracellular bacterial numbers, this process was still thought to benefit immunity. This finding of enhanced phagolysosomal been replicated in more recent studies [80], [81]. Serum from individuals who received two booster vaccinations with BCG showed high titres of anti-LAM antibodies and was used to pre-treat bacilli prior to monocyte or neutrophil uptake [81]. This serum, but not control serum from non-vaccinated individuals enhanced uptake and restricted growth of ingested BCG [81]. Pre-coating of bacilli with this serum rich in anti-LAM antibodies also led to a significant increase in proliferating and IFN-γ-expressing CD4^+^ and CD8^+^ T cells [81].

Opsonizing antibodies isolated from healthy donors in India were able to restrict the growth of *M.tb* h37Rv in macrophages, and showed enhancement of TNF-α and IL-12 secretion [80]. These antibodies, but not non-opsonizing antibodies, were able to enhance intracellular killing by increasing LAMP-1 and iNOS localization to the phagosome as well as phagolysosome acidification [80]. FcγR^−^/^-^ mice are more susceptible to infection with *M.tb*, and loss of function of the inhibitory FcγRIIb improves TB outcome by enhancing IL-12, granuloma formation and IFN-γ levels [82]. A small pilot study in Ethiopia found the presence of FcγR3b copy-number variations to confer additional risk of developing TB in patients with HIV infection, although similar findings were not evident in another Moroccan cohort [83], [84].

The protective effect of IVIG in TB murine models is dependent on antibody binding to FcR [85]. Removal of Fc region glycosylation nullifies antibody interaction with FcR and abolishes the ability of human IVIG infusion in mice to enhance granuloma formation, lung inflammation and bacillary load in infection with *M.tb* [85]. Antibody opsonization of bacilli for targeted FcR-mediated phagocytosis thus appears to be a major mechanism of protection in models of TB infection.

#### 1.4.3 Other mechanisms of AMI in TB

Although FcR-mediated phagocytosis is likely to be the predominant mechanism of AMI against intracellular pathogens, other pathways for antibody protection have been explored in TB. Complement-induced killing by activation of membrane attack complexes is augmented by antibody opsonisation. Complement and IgG binding of live *M.tb* h37Rv increases the number of viable intracellular bacilli, rather than reduce bacterial load which would be expected if antibody-dependant complement-mediated destruction occurred in TB [86]. This is in keeping with the previously mentioned activation of C1q that could be enhancing uptake of bacilli into their intracellular niche [20].

Antibody dependant cell-mediated cytotoxicity has not yet been described to exist through the action of antibodies against *M.tb*. Antibodies are able to directly neutralize a broad range of bacterial and viral pathogens, but little evidence exists for this action of antibody against mycobacteria. However, yeast killer toxin–like antibodies are able to directly bind to and kill a multi-drug resistant strain of *M.tb* [87]. These antibodies were stimulated either by natural candida infection, or by anti-idiotype inoculation of mice with subsequent hybridoma formation [87]. More antibodies with direct mycobactericidal properties have been described, and thus it seems unlikely that the human immune response produces potently neutralizing antibodies capable of killing or inhibiting the growth of mycobacteria independently of phagocytes.

#### 1.4.4 Mucosal protection against TB

Another potential role for antibodies against TB could be to modify the initial encounter with pathogen in the mucosal lining. Mice lacking the polymeric Ig receptor which transports IgA into the respiratory mucosa, are more susceptible to *M.tb* infection than wild-type mice [88]. This effect is believed to be mediated by immune exclusion and appears similar to findings in *Salmonella typhimurium* infection where this same pIgR knockout increases susceptibility to infection [89]. Secreted IgA is able to prevent lung infection with intranasally inoculated *Shigella flexneri*, a facultative intracellular pathogen, by immune exclusion and removal of bacteria by mucociliary transport [90].

In an experiment demonstrating both the notion that antibodies may prevent cell adhesion of mycobacteria and that specificity is key to this process, Choudhary et al. [91] established that monoclonal and polyclonal antibodies directed only against surface antigens of *Mycobacterium leprae* were able to inhibit cell adhesion to Schwann cells. Thus entry of mycobacteria into host cells may be prevented by the action of antibody. Humans produce high titres of IgM against mycobacterial heparin-binding hemagglutinin (HBHA), a surface expressed molecule that facilitates host cell invasion [92], [93]. Sera from such patients were able to prevent the entry of *M.tb* into an alveolar epithelial cell line [92].

In a BCG model, both IgA knock-out mice and wild-type mice were immunized with aerosolized mycobacterium surface antigen PstS-1 [94]. Mice unable to produce IgA were still able to produce antigen-specific IgG and IgM, but had higher CFU numbers in the lungs at 4 weeks post-infection. The IgA^−^/^-^ mice also had decreased TNF-α and IFN-γ production [94]. The role of IgA against intracellular pathogens is not exclusive to mycobacteria. Aerosolized *Francisella tularensis* live vaccine strain in IgA^−^/^-^ mice demonstrates similar findings to those in *M.tb* with attenuated markers of CMI and worse outcome [95].

In a novel experiment, polyclonal human secretory IgA (hsIgA) was purified from colostrum donated by healthy women, and shown to contain IgA able to bind whole BCG and *M.tb* lysate [96]. Prophylactic intratracheal incubation or pre-incubation of *M.tb* with this hsIgA reduced bacillary load and improved granuloma formation in the lungs of mice challenged with live *M.tb* [96]. This showed that antibody capable of interacting with *M.tb* in the mucosa may be passively passed on between mother and child, and that human *M.tb* specific hsIgA can modify the course of infection [96].

### 1.5 Monoclonal antibodies against TB

The study of monoclonal antibodies (mAbs) has been useful in further dissecting the role of humoral immunity. The first mAbs against mycobacteria were described in the 1980s where hybridomas were used to generate antibodies against *M.tb* H37Rv, *Mycobacterium bovis BCG* and *M. leprae* [97]. It was observed that certain mAbs bound to species-specific epitopes [97]. Giving credence to the theory that antibodies can opsonize mycobacteria, Glatman-Freedman et al. isolated 3 mAbs from mouse hybridomas that were able to bind epitopes on the surface of *M.tb* as confirmed by EM [98].

These mAbs were subsequently tested in a mouse model of infection, where only the 9d8 antibody, an IgG3 specific for arabinomannan, was able to prolong survival after lethal dose challenge via enhancement of granuloma formation and iNOS localization to cells containing *M.tb* [99]. Of note is that the other two mAbs, both of the IgM isotope, were not able to enhance survival despite their validated surface binding, confirming that antibody class likely plays a role in conferring protection [99]. The 9d8 mAb was then tested in IFN-γ and MHC class II knock-out mice, where it was less efficacious at improving protection, suggesting interaction with CMI is required to confer protection [99]. A mAb able to bind the AM moiety of LAM on the surface of *M.tb* (SMITB14) led to improved weight loss, bacillary load and survival in *M.tb* challenge when given IV prior to infection as well as when bound to bacilli upon inoculation [100].

HBHA facilitates entry of *M.tb* into epithelial cells and is required by the bacteria to disseminate in mouse models of infection [101]. Pre-coating of BCG with anti-HBHA mAbs prior to intranasal inoculation did not influence bacterial CFU in lungs, but led to a marked reduction in spleen colonization [101]. This finding supports previously described studies suggesting that antibodies directed against LAM prevented dissemination of infection and that anti-HBHA IgM is able to prevent epithelial cell invasion [92], [102]. However Parra et al. [103] could not replicate the protective effects of an anti-HBHA mAb against disseminated disease in a virulent strain of *M.tb* contrary to previous findings with BCG.

#### 1.5.1 IgA mAbs in passive immunotherapy

Other mAbs have been used in attempts to assess the possibility that IgA in the airway protects against TB as suggested by studies with mice lacking pIgR or the transfer of hsIgA above [89], [95]. Several studies have confirmed the plausibility that IgA directed against Acr may confer passive protection [104], [105], [106]. Lopez and Williams et al. [105], [106] have shown that intratracheal or intranasal inoculation with murine IgA against Acr reduces bacillary load and improves granuloma formation. This reduction in CFU occurred early in intra-tracheal inoculation and after 3 weeks post intranasal administration [105], [106]. Both groups compared the functional IgA antibody to antibodies against the 38 kDa antigen, and antibodies of the latter specificity were found to be non-functional, again showing that antibodies against *M.tb* vary in protective function according to epitope [103], [104].

Later, a single chain variable antibody fragment specific for Acr was generated using a phage display library and then expressed in CHO cells with a human IgA1 constant region [104]. This antibody was tested in mice expressing a transgenic CD89 where it demonstrated the same type of complex *in vivo* action observed for the 9d8 antibody, where the therapeutic effect is greater *in vivo* than *in vitro*. The chimeric IgA antibody was able to reduce bacterial load when co-administered with IFN-γ, and only in the presence of the transgenic CD89 receptor, suggesting a receptor-mediated effect with interaction with IFN-γ stimulation pathways [104].

Another surface-binding murine mAb against MPT51 is able to agglutinate cultures of the pathogenic strain CDC1551 at a concentration of 100ug/ml in comparison to a control anti-*M.tb* mAb that did not bind the surface of this strain [107]. This corroborates findings that antibodies can interact with inhaled mycobacteria in the mucosa, and may be a mechanism whereby antibodies prevent entry into host cells.

Although these studies have limitations in methodology, they form a coherent picture (summarized in Table 2) that antibodies contributing to immunity against *M.tb* are likely those that opsonize the bacillus and are able to modulate the host macrophage response via targeting bacilli to FcR–mediated phagocytosis. In addition, immune exclusion may be a potential mechanism whereby antibodies could prevent binding of mycobacterial adhesins such as HBHA to host cells or cause mycobacterial clumping. This may limit dissemination of infection or prevent entry of *M.tb* into cells in the lung mucosa.

### 1.6 Future directions for antibody research in TB

#### 1.6.1 Passive immunotherapy

Interest in immunotherapies against *M.tb* has been galvanized by the increasing rates of drug resistance and HIV co-infection globally [108]. Therapeutic mAbs have been proposed as one potential avenue of exploration to intervene in these clinical scenarios, given the demonstration that several mouse mAbs improve outcome in experimental models of disease [109].

MAbs against the surface of Methicillin Resistant Staphylococcus Aureus (MRSA) have very recently been used to deliver antibiotic directly to host cells, where MRSA appears to establish intracellular reservoirs to evade host immunity [110]. Using similar novel antibody-antibiotic conjugate technology, mAbs could potentially be adapted to deliver drugs directly to TB-infected host cells.

#### 1.6.2 Cell-mediated immunity may not be sufficient to confer protection by vaccination

Recent studies have cast doubt on the sufficiency of MHC-II restricted CD4^+^ cells to confer protection by vaccination. BCG-specific CD4^+^, CD8^+^ and γδ T-cell expression of IFN-γ, TNF-α, IL-2, and IL-17 do not seem to correlate with the protection conferred by BCG in infants [111]. However in a more recent study, BCG-specific ELISPOT responses are associated with a reduced risk of disease in BCG-vaccinated South African infants [37]. BCG-specific T-cell ELISPOT antigen specific CD4^+^ T-cells do not appear to localize to the site of infection in lung tissue until 18–20 days after the establishment of disease, showing that *M.tb* delays the onset of adaptive Th1 immunity [112]. In contrast, several mAb studies demonstrate an early impact on CFU in *in vitro* models. Other arms of the immune system such as T-cells of the γδ, CD1-restricted, Th17 and mucosal associated innate T-cells subsets have a demonstrable role in protection and may be able to circumvent these limitations [6]. However, no prior vaccine has attempted the induction of such a memory T-cell response.

Thus exploring whether AMI may boost vaccine-induced immunity offers several conceptual advantages to future vaccination design. Firstly, antibodies have the potential to engage with the initial inoculum of bacilli in the mucosal surface, and can thus potentially influence host immunity earlier than CD4^+^ T-cell responses. Secondly, this offers a clearer path to measuring, at least in part, one correlate of protection if protective mAbs can be found. Thirdly, this approach fits with current models of immunology where antibody and CMI are synergists in conferring protection against intracellular pathogens. A consistent finding of the protective antibodies detailed is an enhancement of cytokine production (TNF- α, IL-12), activation of T-cells and granuloma formation in *in vivo* models. This suggests that a vaccine incorporating the induction of protective antibodies may also augment the development of an early and effective T-cell response against *M.tb*

#### 1.6.3 Models of vaccines that incorporate the induction of antibodies in TB

Such vaccines that induce both antibody and cellular responses have been examined in animal models. Membrane vesicles from H37Rv induce antibodies presumably to surface lipoproteins, as well as poly-functional CD4^+^ cells, and are able to elicit protection superior to live BCG in a mouse model [113]. Arabinomannan-protein conjugate vaccination in mice induced both antibody and T-cell responses, and the protective effect was thought to be in part be due to antibodies that function similarly to the 9d8 antibody described above [114]. Counteraction of LAM-induced T-cell suppression and inhibition of macrophage function by antibody is another potential mechanism for this finding [114]. Aerosolized BCG vaccination induces an IgG as well as CMI in rhesus macaques and improves the protection provided by intradermal BCG injection [115]. In humans, orally administered BCG is able to induce the formation of anti-LAM secreted IgA in the respiratory mucosa, however the efficacy of this approach has yet to be determined [116].

Antibody specificity may be key to these observed effects, as no protective role for B-cells was found in mice vaccinated with aerosolized Adhu5Ag85A, the same antigen as the MVA85A vaccine [117]. Of note is that aerosolized vaccination induced CD4^+^ and CD8^+^ responses as well as IgG and IgA in the BAL fluid against Ag85A [117]. Rather than hinder further testing of vaccines to induce both AMI and T-cell responses, such a study highlights the need to identify the epitopes that are likely to stimulate functional antibodies against *M.tb.* It is encouraging for vaccine design that mucosal vaccination can induce antibody responses both systemically and in the lung mucosa, and the induction of antibodies capable of opsonizing *M.tb* by aerosolized vaccination merits testing in similar models.

#### 1.6.4 Potential directions for future antibody research in TB

The lack of data concerning the epitopes of functional antibodies must be addressed in order to test the contribution of such antibodies to vaccine-induced immunity. Advances in the methods of mAb production have recently led to the ability to rapidly clone large libraries of mAbs directly from patient-derived plasmablasts or memory B-cells [118], [119]. Future work might therefore include the production of human mAbs (hmAbs) against *M.tb*. Proof of this concept is the discovery of protective hmAbs against a diverse range of pathogens including malaria, pneumococcus, influenza and HIV [118], [120], [121], [122]. A novel envelope dimer epitope of dengue virus was identified by characterizing hmAbs from infected patients, and this antigen appears promising for downstream vaccine translation Further, the 3BNC117 and VRC01 hmAbs, cloned from patients infected with HIV, have recently seen use as intravenous therapeutics capable of reducing viral load in HIV-1 infected patients [123], [124]. HmAb cloning therefore present a potent new tool that may aide the discovery of protective antibodies against *M.tb* from humans, and point to novel antigens to induce such antibodies by vaccination.

Next-generation sequencing has also contributed to the elucidation of the antibody repertoire in response to infection and vaccination [125], [126]. These advances may allow mapping of how the antibody response develops during active TB infection, and how it is subverted by *M.tb* to prevent the formation of any protective antibodies as suggested by the antigenic variation in B-cell epitopes and the lack of surface-binding antibodies. The study of clinical populations who appear to be resistant to acquiring latent infection with *M.tb* are also of great interest in order to understand immunity against TB [37]. There is strong suggestion that healthy individuals produce high titres of antibodies to PPD and Ag85A upon exposure to mycobacteria, and studies to assess the effect of antibody responses on preventing active or latent infection are a promising avenue of exploration. Ultimately, there is a need to better understand the role for antibodies in human immunity such that the ability of antibodies to limit dissemination of *M.tb* and target bacilli for FcR-mediated phagocytosis can be harnessed by vaccination.

## 2 Conclusion

Infection and disease caused by *M.tb* strongly stimulates humoral immunity in humans. Although CMI remains the predominant correlate of protection, there is evidence to suggest that antibodies may contribute, at least in part, to immunity. The presence of antibodies against specific *M.tb* antigens such as LAM appears to differ in patients with pulmonary and disseminated TB. This corresponds to mAbs against LAM and HBHA that are able to decrease bacillary load and prevent dissemination of mycobacterial infection. Antibodies are now widely understood to modulate CMI through FcR binding, and particularly surface-binding antibodies in cases of mycobacterial infection in experimental models. It is thus of interest that such antibodies do not seem to be stimulated during natural infection, and the trend in protective mAbs thus far is a suggestion that surface binding with targeting to FcR for enhancement of CMI can occur *in vivo.*

These findings point to the need for further testing of whether antibodies may confer superior protection in vaccination by enhancing CMI, or are able to prevent infection if present prior to host encounter with *M.tb*. Many challenges stand in this path, such as a lack of knowledge concerning the existence of functional mAbs in humans and which epitopes are likely to induce their formation. However, new technologies now exist to conclusively address the feasibility of incorporating goals to target AMI in the design of next-generation vaccine candidates.

## Funding

None.

## Competing interests

None declared.

## Figures and Tables

**Table 1:** Factors related to the development of antibodies against *M.tb* in humans.

Active disease	Latent infection	Effect of anti-TB treatment	Bacterial factors	HIV co-infection	Host genetics
Hypergammaglobulinemia in 90% of patients [14]	Range of antibodies may reflect antigens produced by *M.tb* during latency *e.g*. Acr [44]	Broader range of antigens recognized possibly due to antigens presented from killed bacilli [127]	Expression of antigens vary in different strains of *M.tb* and may influence humoral responses [128]	Greater homogeneity in IgG subclass recognizing LAM. IgG1 and IgG4 produced against LAM but not IgG2 [102]	Association with HLA-DR15 to development of active TB and higher anti-38 kDa antibody levels [129]
Raised mycobacteria-specific plasmablasts and plasma cell counts in peripheral circulation [130]	In high exposure settings, high titres of antibodies against CFP-10 and ESAT-6 are frequently seen in latency [131]	Gradual decrease in antibody titres as active disease resolves [132]	Bacterial expression of antigens during replication and dormancy may influence humoral responses [132]	Recognition of certain antigens such as MPT51 correlate stronger to active TB disease in HIV-1 infected persons than non-infected individuals [133]	HLA-DR2 and HLA-DQw1 associated with recognition of the TB71 and TB72 epitopes of the 38-kDa antigen [129]
Sputum smear-positive disease associated with higher titres of antibodies than other forms of disease [129]				Prevalence of patients with IgG against PPD decreases with lower CD4+ counts [39]	

**Table 2:** Evidence suggestive of a role of antibody-mediated immunity in prevention of infection or limiting severity of disease.

	Prevention of infection	Limiting severity of disease	Mucosal immunity
Clinical observation	• High titres of IgG against PPD seen in persistently exposed but TST- negative individuals [45] • Strong antibody responses against *M.tb* surface in healthy controls from high burden country [47]	• Lack of antibodies against LAM, membrane vesicles and 38-kDa antigen associated with risk of extra-pulmonary disease [34], [36], [44] • Higher titres of IgG against Ag85A associated with reduced risk of disease in infants [37]	• IgA against HrpA associated with improved severity markers on presentation in active TB disease [40] • Oral vaccination with BCG is able to induce anti-LAM sIgA in respiratory mucosa [134]
Experimental models	• No studies available to demonstrate that antibodies prevent acquisition of infection with *M.tb*	• Serum enriched for antibodies against LAM enhances intracellular killing of *M.tb* [81] • IVIG improves granuloma organization and decreases lung CFU in mouse models [85]	• IgA^KO^ and pIgR^KO^ mice more susceptible to *M.tb* [88], [94] • Prophylactic intra-tracheal human secretory IgA from colostrum can protect mice against *M.tb* challenge [96]
Monoclonal antibodies	• MAb against MPT51 agglutinates cultures of *M.tb* CDC1551 and may show immune exclusion mechanism [107]	• Intravenous Anti-LAM mAb enhances granuloma formation and prolongs survival in mice [100] • Anti-HBHA mAb limits dissemination of BCG in mice [103]	• Passive immunotherapy with IgA mAb against Acr of protective benefit in mouse model [104]
